# Supplementary material for: Synergistic Effects of Ladder and Cage Structured Phosphorus-Containing POSS with Tetrabutyl Titanate on Flame Retardancy of Vinyl Epoxy Resins
Source: Polymers (Basel). 2021 Apr 22;13(9):1363. doi: 10.3390/polym13091363 (PMC8122454; doi:10.3390/polym13091363)
Supplement: Supplementary file 1 [file polymers-13-01363-s001.zip › polymers-1168253-supplementary.pdf]

Synergistic effects of ladder and cage structured phosphorus-containing POSS with tetrabutyl titanate on flame retardancy of vinyl epoxy resins

Xu Han[a,b], Xiaohua Zhang[c], Ying Guo[b], Xianyuan Liu[b], Xiaojuan Zhao[b,*], Heng Zhou[b,*], Songli Zhang[a,] Tong Zhao[b]**

[a] School of Materials Science and Engineering, Jiangsu University, Zhenjiang, 212013, China

[b] Key Laboratory of Science and Technology on High-Tech Polymer Materials, Institute of Chemistry, Chinese Academy of Science, Beijing, 100190, China

[c] Beijing University of Chemical Technology, Beijing, 100029, China

Correspondence

[*]Heng Zhou, Key Laboratory of Science and Technology on High-Tech Polymer Materials, Institute of Chemistry, Chinese Academy of Science, Beijing, 100190, China

Email: zhouheng@iccas.ac.cn

[*]Xiaojuan Zhao, Key Laboratory of Science and Technology on High-Tech Polymer Materials, Institute of Chemistry, Chinese Academy of Science, Beijing, 100190, China

Email: zhaoxj@iccas.ac.cn

[**]Songli Zhang, School of Materials Science and Engineering, Jiangsu University, Zhenjiang, 212013, China

Email: 157807042@qq.com

Index of Figure and Table

FIGURE S1 FTIR spectra of DOPO (a), VTES (b) and DOPO-VTES (c)

FIGURE S2 The ^{1}H-NMR spectra of DOPO (a), VTES (b), DOPO-VTES (c)

FIGURE S3 FTIR spectra of DOPO -VTES (a), DOPO-POSS (b)

FIGURE S4 ^{1}H-NMR spectra of DOPO -VTES (a) and DOPO-POSS (b)

FIGURE S5 ^{29}Si-NMR spectra of DOPO-POSS

FIGURE S6 XRD pattern of DOPO-POSS

FIGURE S7 MALDI-TOF MS spectrum of DOPO-POSS

FIGURE S8 The curves of storage modulus and tan δ versus temperature of the VE composites

TABLE S1 DMA data of the VE composites

FIGURE S9 XPS spectra of Si2p (b), P2p (c), C1s (d), O1s (e) spectra for VE-2

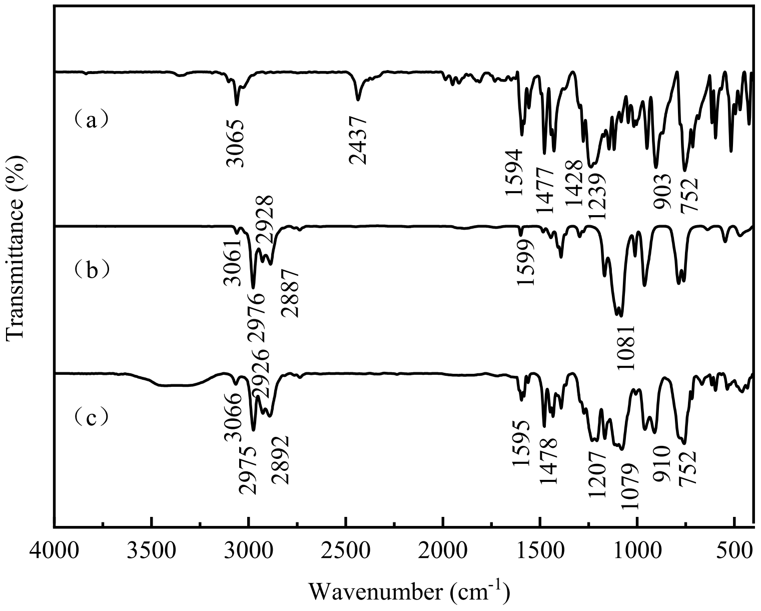

FIGURE S1 FTIR spectra of DOPO (a), VTES (b) and DOPO-VTES (c)

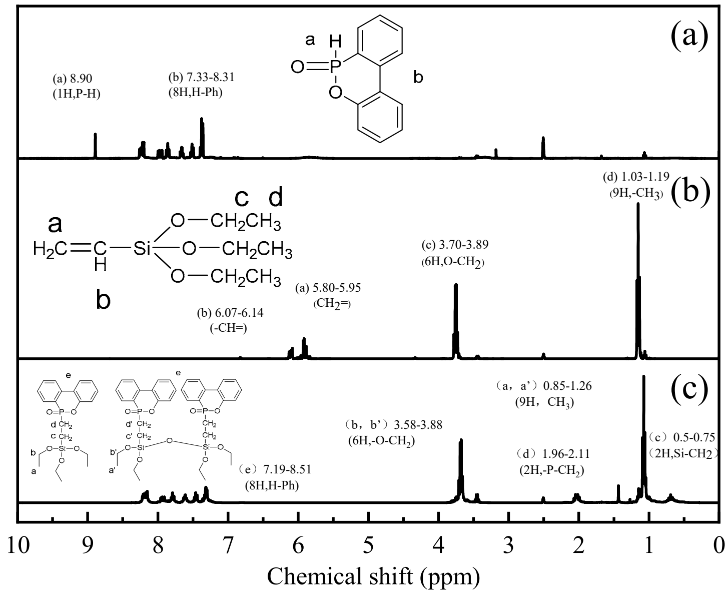

FIGURE S2 The ^{1}H-NMR spectra of DOPO (a), VTES (b), DOPO-VTES (c)

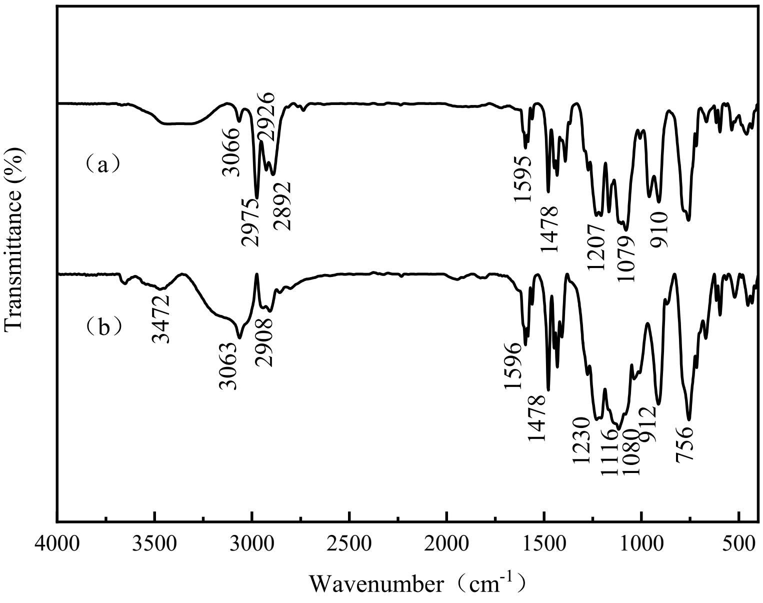

FIGURE S3 FTIR spectra of DOPO -VTES (a), DOPO-POSS (b)

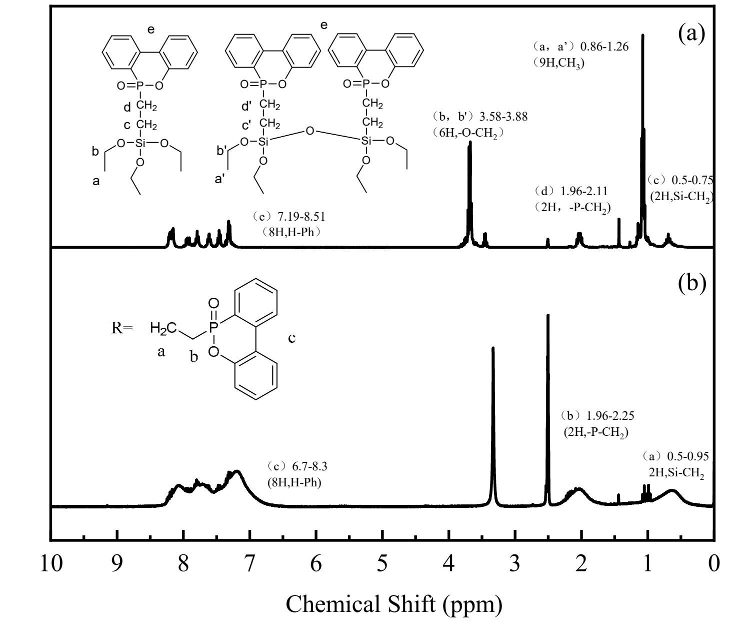

FIGURE S4 ^{1}H-NMR spectra of DOPO -VTES (a) and DOPO-POSS (b)

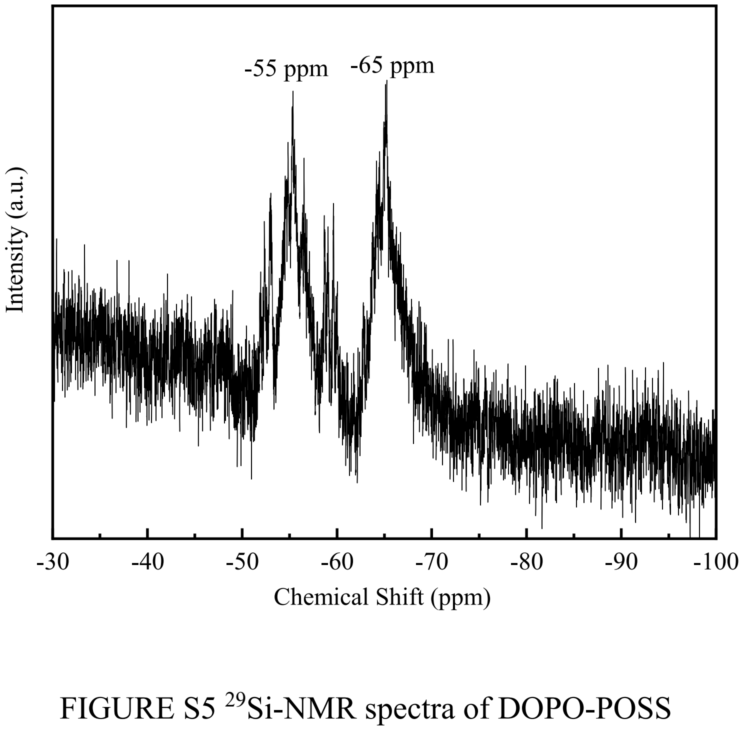

FIGURE S5 ^{29}Si-NMR spectra of DOPO-POSS

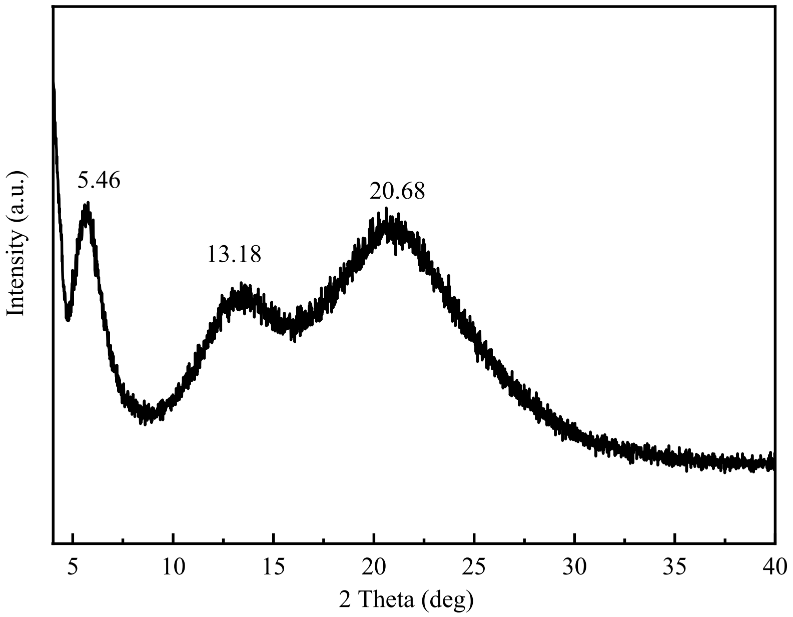

FIGURE S6 XRD pattern of DOPO-POSS

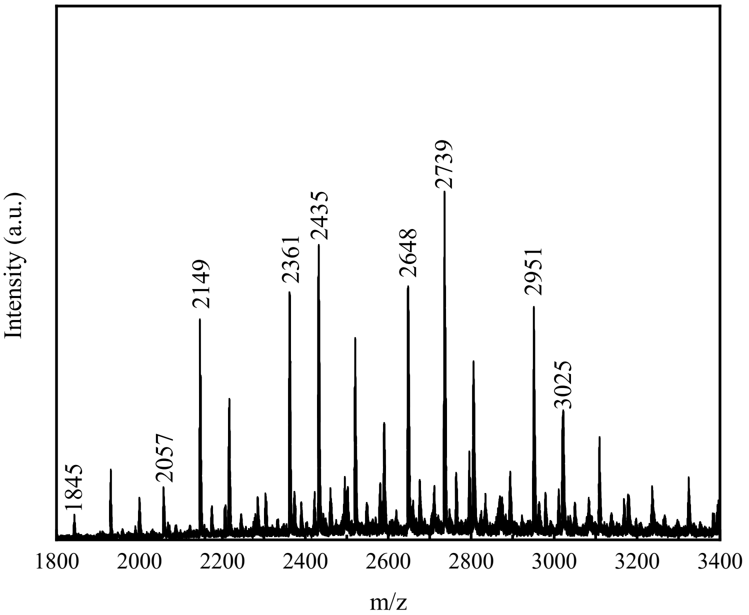

FIGURE S7 MALDI-TOF MS spectrum of DOPO-POSS

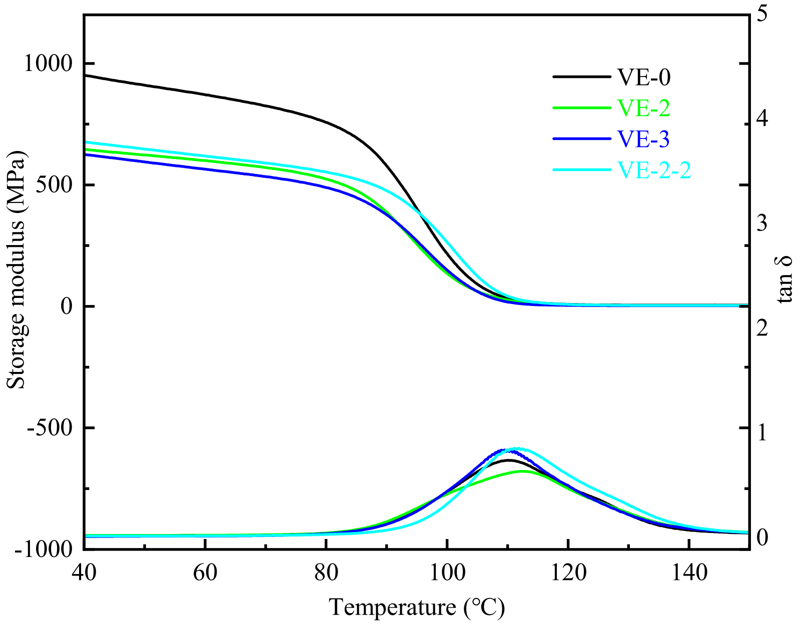

FIGURE S8 The curves of storage modulus and tan δ versus temperature of the VE composites

TABLE S1 DMA data of the VE composites

Samples	$E'_{40^{\circ}C}$ (MPa)	$E'_{150^{\circ}C}$ (MPa)	T_g (°C)
VE-0	951.11	4.97	110.06
VE-2	646.10	3.07	112.81
VE-3	625.11	2.60	109.85
VE-2-2	675.69	3.66	111.43

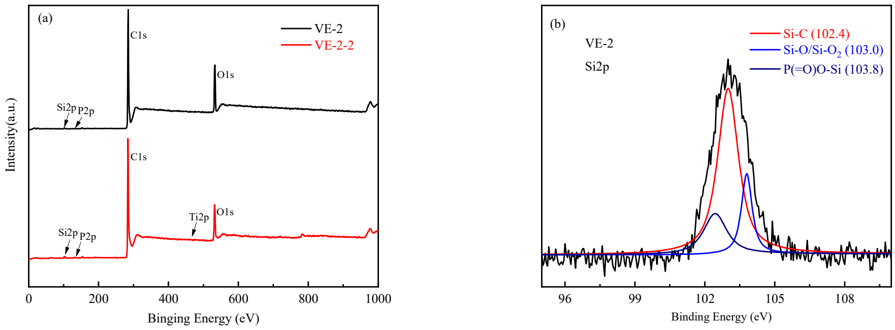

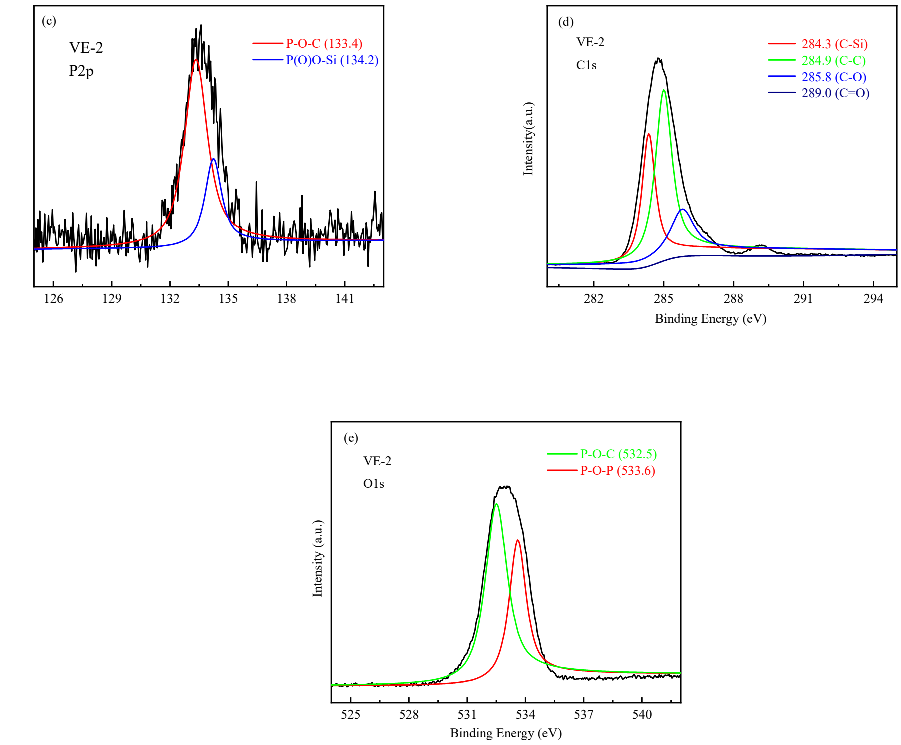

Figure S9 XPS spectra of Si2p (b), P2p (c), C1s (d), O1s (e) spectra for VE-2
